# Renal cell carcinoma presenting as a solitary cutaneous facial metastasis: case report and review of the literature

**DOI:** 10.1186/1477-7800-3-27

**Published:** 2006-09-12

**Authors:** Neil A Porter, Helen L Anderson, Saad Al-Dujaily

**Affiliations:** 1 Department of Urology, Basildon University Hospital, Essex, UK

## Abstract

**Background:**

Renal cell carcinoma is well known for its frequency to metastasise, particularly to lungs, liver, bones and brain. Metastasis to the skin is much less common. Presentation as a result of the skin lesion is even more unusual, with only 14 previously reported cases in the English literature. The majority of these cases have been reported in patients with recurrent disease or with other metastases.

**Case presentation:**

We present only the second case of non-recurrent renal cell carcinoma with a solitary cutaneous facial metastasis reported in the English literature.

**Conclusion:**

Clinicians should conduct a careful inspection of the skin in patients with renal cell carcinoma and also have a high index of suspicion of primary internal organ malignancy in patients presenting with a skin lesion.

## Background

Renal cell carcinoma (RCC) accounts for 3% of all adult malignancies [1]. Presentation with the classic triad of flank pain, haematuria and a palpable abdominal mass is uncommon. Most present with only one of the above symptoms or as an incidental diagnosis as a result of radiological imaging for another reason. Some patients may present as a result of symptoms from secondary metastases.

Metastasis of RCC to organs such as the lungs, liver, bones and brain is well known, but there are far fewer reports of metastases to the skin. Cutaneous lesions as the primary presentation of renal cell carcinoma are extremely uncommon with only a handful of reports in the literature. We report a case of RCC presenting as a solitary cutaneous facial metastasis with no other organ involvement and no past history of RCC. This is only the second such case to be reported in the English literature (although no details of other organ involvement were documented in the previously reported case) and may be the first case presenting as a facial abscess.

## Case presentation

**Figure 1:**
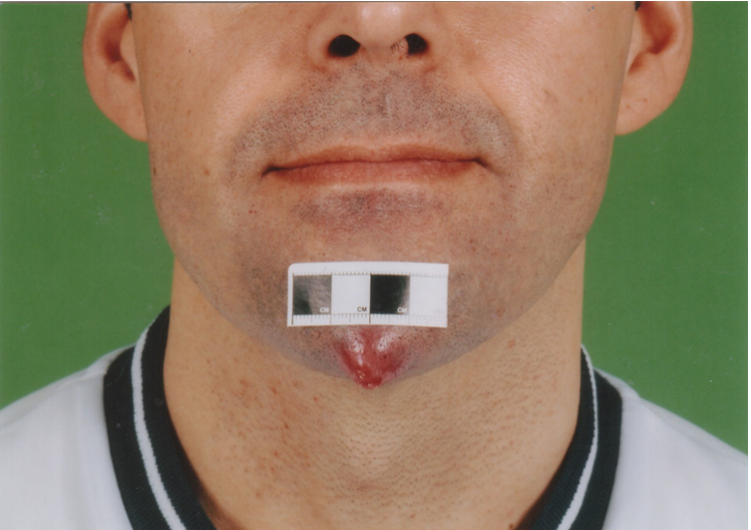
View of the patient's face showing the renal cell carcinoma metastasis on his chin.

**Figure 2:**
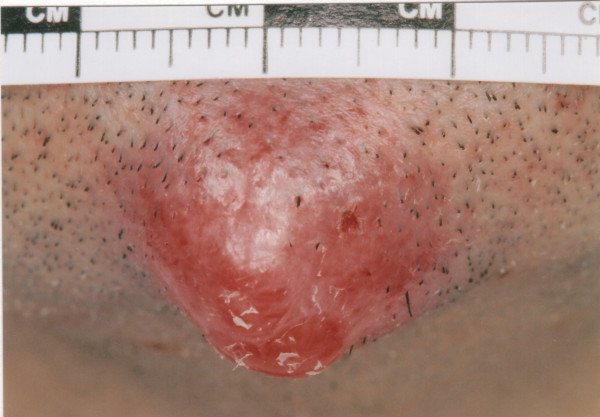
Close-up view of the metastasis on the patient's chin.

A 36 year old male was referred by his general practitioner to the accident and emergency department with what was thought to be an abscess on his chin (Figures 1 and 2). The lesion had been present for eight weeks and had not improved despite a six week course of antibiotics from his general practitioner. The lesion had increased in size over the last two weeks and at one point the patient noticed it had bled slightly whilst he was shaving. There had been no other discharge.

During the two weeks before presentation the patient had been feeling generally unwell with fatigue and lethargy. He also reported weight loss of about 5 kg over the last month.

On further questioning he described some left sided dull, 'dragging' abdominal pain and associated back pain. He denied any change in his bowel habit and had no haematuria or other urinary symptoms. He had no past medical history and was not on any medications. He was a non-smoker.

On examination the patient was lethargic with a prominent lesion on his chin 2 cm in diameter. The lesion was erythematous, tender, tense and was similar to the appearance of an abscess. He was afebrile.

On examination of the abdomen he was found to have a number of purpuric spots over the upper torso and hepatosplenomegaly.

Investigations showed normal full blood count, urea and electrolytes, liver function tests and chest X-ray. His C-reactive protein was elevated at 155.

In view of his weight loss and hepatosplenomegaly he was admitted under the medical team for further investigation with a suspicion of lymphoreticular malignancy.

The surgical team were asked to review regarding the "abscess" on the patient's chin. However, due to the pending investigations a decision was made to wait for the results before contemplating incision and drainage.

The patient had a computed tomography (CT) scan of the abdomen and pelvis which diagnosed a left renal carcinoma 12 cm in diameter arising from the anterior cortex, but no thrombus or tumour was identified in the left renal vein. The patient was then referred to the urology team.

The urology team arranged a chest CT scan and a bone scan which were normal and subsequently performed left radical nephrectomy and biopsy of his chin lesion.

The histology of his left kidney showed clear cell renal carcinoma, Stage 3, with tumour invading the left renal vein at the kidney hilum (G3 pT3b N0 M1).

Malignant cells were found in the chin biopsy histology, which were consistent with metastatic carcinoma.

The patient was referred to the oncologists for further treatment. He underwent subcutaneous interferon injections at a dose of 5 mega units three times per week for a total of 4 months. He also underwent radiotherapy to the site of the lesion on his chin, followed by resection of the lump. One year later the patient developed some pain in his iliac crest and right posterior ribs for which he underwent palliative radiotherapy although bone scan and X-rays did not show any evidence of metastasis.

It is now two and a half years since diagnosis and the patient remains disease free, having had a recent follow up CT scan which showed no evidence of recurrence.

## Discussion

Renal cell carcinoma (RCC) has been well described for its frequency to metastasise, occurring in approximately one third of patients at the time of diagnosis [2]. However metastases from RCC to the skin are much less common, more usual sites being the lung, lymph nodes and bones [3]. One large study of 6577 autopsies found 54 cases of unrecognised RCC and documented that the skin was the 7^th ^most common site of metastasis [4]. RCC is the primary tumour in approximately 6% of skin metastasis [5,6].

There have been several previously reported cases of RCC metastasising to the skin although there are only a handful of reports of RCC presenting with skin metastasis.

In our search through the literature (on PubMed using the keywords renal cell carcinoma skin metastasis) we found only 14 other cases of non-recurrent renal cell carcinoma presenting with skin metastasis reported in English [8-12] and 1 reported in French [13].

The only previously reported case in the English literature of a patient with non-recurrent RCC presenting with a solitary facial metastasis is of 63 year old male with a solitary chin metastasis reported in 1978. No details of other organ involvement or outcome were provided in the article [8].

A Japanese literature review reported 75 cases of RCC associated with skin metastasis. The commonest site of metastasis was to the trunk (40%), followed by the scalp (25%). Only 8% had metastases to the face. 24% had cutaneous metastases at the time of diagnosis [7]. A further literature review from Japan showed that there have been a total of 22 cases of RCC presenting with skin metastasis, with only one case of a solitary facial metastasis (although the presence of metastases in other organs was not mentioned) [14].

A large Indian study, reviewing a total of 306 patients with RCC seen over a 12 year period found only 10 cases (3.3%) with skin metastases. Of these, half of the patients presented with skin metastases during follow-up after nephrectomy. In only one case the skin nodule was actually the presenting symptom. This patient, a 32 year old male had a nodule located on the chest and also had lung metastases at the time of diagnosis (outcome not documented). 90% of the patients also had metastases to other organs at the time of diagnosis [9].

A Greek study reviewed 6 patients with RCC presenting with skin metastases, although none were to the face. 3 of them had solitary cutaneous metastases, one 76 year old male also had lung metastases, other organ involvement was not documented in the other two cases (41 year old male and 81 year old female). The 3 patients with multiple cutaneous metastases all had other organ involvement at the time of diagnosis. Sites of cutaneous metastasis were scalp, thorax and abdominal wall [10].

A large retrospective study of 130 patients with metastatic renal cancer revealed cutaneous metastases in 6 patients. 2 of these had only local metastases in the previous nephrectomy scar, the other 4 had only distal metastases, 3 of which were to the scalp and one patient had multiple facial, neck, chest and upper extremity metastases. No details regarding other organ involvement or previous RCC were provided in the article [11].

Other authors have reported single cases. A 55 year old male presented with a bleeding metastasis on the tip of the nose, the patient also had liver and lung metastases and died within weeks from a brain haemorrhage [12]. A 74 year old male presented with a 3 cm isolated scalp lesion. He had no other cutaneous metastases and no other organ metastases [13].

There have also been 4 further reports of recurrent RCC presenting with cutaneous metastases after nephrectomy:-

▪ 55 year old male with a solitary metastasis on the chin presented during follow-up, the patient declined further treatment and died 6 months later [3].

▪ 61 year old male with a scalp metastasis and also had bone and chest metastases [15].

▪ 75 year old female presented with a skin metastasis on the back 4 years post nephrectomy. The patient also had lung and bone metastases [16].

▪ 61 year old male with 3 skin lesions in front of left ear. The patient also had liver metastases at time of diagnosis and developed recurrence of the skin lesions post operatively. He died 1 year post-nephrectomy [17].

Several of the above studies have noted the poor prognosis of patients with skin metastases at the time of diagnosis of RCC, often heralding advanced disease. However, in our case the patient remains disease free two and a half years further on. It is likely that the improved outcome is a result of having only a solitary cutaneous metastasis, along with the prompt diagnosis and treatment of the underlying primary malignancy.

## Conclusion

Metastasis at the time of diagnosis frequently occurs in RCC, with the skin not infrequently involved. RCC may present as a result of symptoms from metastases, including the skin, although solitary cutaneous metastases as a primary presentation are uncommon.

Clinicians should be aware of the possibility of primary internal organ malignancy in patients presenting with cutaneous lesions and therefore conduct a careful examination of the skin in patients with RCC. Prompt diagnosis and treatment may affect the eventual outcome.

## Competing interests

The author(s) declare that they have no competing interests.

## Authors' contributions

NP carried out the literature search and drafted the manuscript. HA summarised the case notes and assisted with the literature search and drafting of the manuscript. SA-D conceived of writing the case report, obtained the notes and photographs and assisted with drafting the manuscript. All authors read and approved the final manuscript.
